# Mapping of Pre-Existing Humoral Immunity to AAV and Natural AAV Capsid Presence in Human Tissues: Observations from a Polish Surgical Cohort

**DOI:** 10.3390/v18101134

**Published:** 2026-10-13

**Authors:** Aleksandra Majewska, Klara Weronika Siejda, Maciej Kowalec, Karolina Aleksandrowicz, Yuliia Panchuk, Małgorzata Marta Rogala, Karolina Pianka, Aleksandra Minich, Weronika Jarocka, Maciej Walędziak, Katarzyna Komorowska, Andrzej Kwiatkowski, Sylwia Rydzik, Tomasz Syryło, Agnieszka Bajek, Michał Wiłkojć, Leszek Gryszko, Grzegorz Suwalski, Jakub Mróz, Michał Zembala, Jacek Doniec, Klaudia Klarysa Brodaczewska, Leszek Lisowski

**Affiliations:** 1 Laboratory of Molecular Oncology and Innovative Therapies, Military Institute of Medicine—National Research Institute, Szaserów 128, 01-141 Warsaw, Poland; amajewska1@wim.mil.pl (A.M.); mkowalec1@wim.mil.pl (M.K.); kaleksandrowicz@wim.mil.pl (K.A.); ypanchuk@wim.mil.pl (Y.P.); mrogala@wim.mil.pl (M.M.R.); kbrodaczewska@wim.mil.pl (K.K.B.); llisowski@wim.mil.pl or llisowski@cmri.org.au (L.L.); 2 BioMedChem Doctoral School of the University of Lodz and Lodz Institutes of the Polish Academy of Sciences, 21/23 Matejki Street, 90-237 Lodz, Poland; 3 Faculty of Biology, University of Warsaw, Miecznikowa 1, 02-096 Warsaw, Poland; 4 Department of General, Oncological, Metabolic and Thoracic Surgery, Military Institute of Medicine—National Research Institute, Szaserów 128, 01-141 Warsaw, Poland; 5 Faculty of Medicine, University of Warsaw, Żwirki I Wigury 61, 02-091 Warsaw, Poland; 6 Department of General, Functional and Oncological Urology, Military Institute of Medicine—National Research Institute, Szaserów 128, 01-141 Warsaw, Poland; 7 Department of Cardiac Surgery, Military Institute of Medicine—National Research Institute, Szaserów 128, 01-141 Warsaw, Poland; 8 Department of Gynaecology and Oncological Gynaecology, Military Institute of Medicine—National Research Institute, Szaserów 128, 01-141 Warsaw, Poland; 9 Translational Vectorology Group, Children’s Medical Research Institute, University of Sydney, Sydney, NSW 2145, Australia

**Keywords:** gene therapy, AAVs, seroprevalence, neutralizing antibodies

## Abstract

Vectors based on adeno-associated viruses (AAVs) are widely used as a delivery system in gene therapy. However, preexisting immunity against AAV remains a major limitation. This study examines the prevalence of AAV genomes and anti-AAV2 immunity in a Polish cohort of adult patients undergoing planned surgical interventions, which serves as a random sample of the general Polish population. The presence of AAV capsid sequences was analyzed in tissue samples using PCR and endogenous AAVs were detected in 33.95% of samples. Anti-AAV2 IgG antibodies were measured by a single point ELISA and were present in 80.93% of patients. Neutralizing activity was evaluated using an AAV2 transduction inhibition assay in HEK293T cells, revealing a 60% reduction in functional activity by patients’ sera. Tissue AAV PCR-positivity was associated with stronger neutralizing activity although the correlation with seropositivity was not as strong. These results suggest that pre-existing humoral immunity against AAV is common in the Polish adult population, indicating frequent exposure to naturally occurring AAVs. The findings provide important data on pre-existing anti-AAV immunity—a key factor considered in clinical trials and gene therapy development. Furthermore, the identification of naturally occurring AAV variants in human tissues points to the potential use of new sources of capsids with potentially distinct biological properties, which could contribute to the advancement of gene therapies.

## 1. Introduction

Adeno-associated viruses (AAVs) are non-pathogenic viruses that, due to their safety profile and ability to transduce dividing and non-dividing cells, constitute a promising gene delivery system in gene therapy.

AAV was discovered as contaminants of monkey and human adenovirus samples [1]. They are small (~26 nm), non-enveloped viruses, which require simultaneous infection with members of the adenovirus, herpesvirus, or papillomavirus families for effective infection [1]. To date, *n* = 13 AAV serotypes and hundreds of natural variants have been reported [2,3]. The 4.68 kb sequence of single-stranded genome of prototypical AAV2, from which recombinant AAV vectors were derived, was determined based on viral cultures propagated in HeLa cells. It consists of two inverted terminal repeat (ITR) sequences flanking open reading frames (ORFs) that encode non-structural (Rep) and structural (Cap) elements necessary for viral particle replication and assembly [4].

The concept of using AAVs as vectors for gene therapy is developing rapidly. The first EMA-approved AAV-based gene therapy was Glybera (an AAV1-based therapy, approved in the European Union in 2012), which targeted familial lipoprotein lipase deficiency. Unfortunately, this therapy was withdrawn in 2017 due to low clinical implementation, driven by high costs and low clinical efficacy. The drug Roctavian (based on the AAV5 capsid)—effective in treating hemophilia A—was also withdrawn in 2026 for business reasons [5]. The technological maturity and interest in the development of AAV-based therapies is evident by the fact that AAV leads as a vector among all vector-based gene therapy clinical trials currently being conducted [6]. Currently, AAV-based gene therapies are used in hemophilia type B: Hemgenix, (AAV5), Beqvez (AAVrh74var), BBM-H901 (engineered AAV843 capsid; approved in China); spinal muscular atrophy—Zolgensma (AAV9); Duchenne muscular dystrophy—Elevidys (AAVrh74); aromatic L-amino acid decarboxylase deficiency—Upstaza (AAV2); Leber congenital amaurosis—Luxturna (AAV2) and glycogen storage disease type Ia—Genglycos (AAV8; approved by FDA) [6,7,8,9].

The primary challenge facing gene therapies at the clinical level is the very high dose of vectors required to achieve the desired therapeutic response. This largely results from the low efficiency of currently available AAV vectors at targeting clinically important organs and tissues. This not only lowers the safety profile of new experimental therapies, but also contributes to the high cost of treatment, which lowers the broad clinical implementation of these potentially life-saving therapies.

Increasing transduction efficiency may be achieved through the development of next-generation AAVs with improved tropisms for specific targets. One of the strategies to develop novel and improved AAVs involves searching for naturally occurring AAVs with desired tissue tropism, which could either serve directly as clinical AAV variants or could form the starting point for further improvements and modifications using genetic engineering [3,10]. Molecular studies have demonstrated the presence of AAV genomes in numerous human tissues and organs, including liver, spleen, bone marrow, skeletal muscle, brain, and lymphatic tissues, establishing human primary tissue samples as a rich source of AAVs with potential clinical applications [11,12]. PCR-based analyses indicate that the frequency of AAV genome detection may vary significantly between different tissues and populations studied, suggesting the existence of specific viral reservoirs in the human body [11]. Therefore, searching for naturally occurring AAVs and using their properties that promote effective infection of specific tissues and organs offers a promising approach to developing more efficient gene therapy delivery systems, reducing the cost and increasing safety of future therapies.

The second major limitation of the current generation of AAV vectors is the preexisting immunity against their protein capsid. This adaptive immunity (particularly humoral in the form of neutralizing antibodies—NAbs) against wild-type AAVs poses a significant challenge to efficient systemic gene therapeutics delivery using these vectors. It is estimated that up to 80% of the human population may have anti-AAV antibodies, with the exact percentage of sero-positive population depending on the AAV serotype and the analyzed cohort [13,14]. For example, Calcedo et al. demonstrated the presence of neutralizing antibodies against different AAV serotypes in 20–70% of population from North America, Europe, and Asia, with the highest seroprevalence observed for AAV2 [15]. The percentage of subjects with detectable levels of neutralizing antibodies is also age-dependent. A study that examined the presence of anti-AAV antibodies against a bioengineered AAV-LK03 variant in the UK population reported that the lowest seroprevalence was observed in the cohort among the 6–14-year-olds, while the highest rate of seroprevalence was observed in late adulthood (43–84 years old) [16]. The level of homology between individual AAV serotypes ranges from approximately 50 to 99%, leading to significant immune cross-reactivity, where antibodies raised against one AAV serotype or variant frequently recognize also other AAVs. This high frequency of the presence of pre-existing antibodies among the patient population and high level of immune cross-reactivity between antibodies against individual AAV variants, including bioengineered variants, unfortunately translates into the exclusion of many patients from clinical trials, thus limiting the impact of current AAV technologies on healthcare and society.

Due to large disparities between populations/geographic regions and insufficient data on the Central and Eastern European population, our study aimed to evaluate the presence of anti-AAV antibodies in the Polish adult surgical cohort. Furthermore, access to tissue samples from serum donors, allowed us also to evaluate the prevalence of the AAV genome in various human organs and correlate the presence of pre-virus with seroreactivity and ability to neutralize AAV transduction. Investigating both the assessment of AAV occurrence in tissue and the level of immune response can provide deeper insights into the biology and epidemiology of AAV, which may contribute to the better efficacy of AAV-based gene therapies.

## 2. Materials and Methods

### 2.1. Study Population

Tissues and blood samples were collected between November 2025 and April 2026 from adult patients undergoing planned medical procedures performed in the clinics of the Military Institute of Medicine—National Research Institute in Warsaw, Poland. Informed consent was obtained from all subjects involved in the study. The study was approved by the Bioethics Committee of the Military Institute of Medicine—National Research institute (17 September 2025, decision no. 44/25). The study included 49.77% men and 50.23% women, aged 19 to 79 years (mean 55.75 ± 16.12; median 58).

### 2.2. Tissue Samples

Stomach fragments (*n* = 99) were obtained from patients undergoing bariatric surgery. Kidney fragments (*n* = 46) were obtained during renal tumor removal procedures (with a healthy margin) and biopsies (planned diagnostic procedures for patients with kidney diseases). Lung fragments were obtained during lung resection procedures (*n* = 24). Heart fragments (right atrial appendage, *n* = 43) and fragments of blood vessels (internal thoracic artery, *n* = 33) were obtained during coronary artery bypass surgery. In some cases, both heart and blood vessels samples were obtained from the same person (*n* = 30). Tissue fragments, used in this experiment, constituted medical waste from the medical procedure performed. Tissue fragments were portioned into ~20 mg pieces and frozen at −80 °C until analysis.

### 2.3. Serum Samples

Blood samples were collected from the same patients from whom tissue fragments were obtained (total *n* = 215). Peripheral blood was drawn into a serum tube (Cat# 367955, Vacutainer SST™ II Advance, BD, Warsaw, Poland) and allowed to clot for 30 min at room temperature (RT). To obtain serum, blood samples were fractionated by centrifugation (2000× *g* for 15 min at RT), supernatants were aliquoted, frozen, and stored at −80 °C. Due to high haemolysis, 6 samples were not included in further assays (total sera *n* = 209).

### 2.4. DNA Isolation

The choice of DNA isolation method depended on both the amount of collected tissue material and the DNA extraction efficiency observed for the individual tissue types (choice of methods was optimized in preliminary experiments; for screening one methods of isolation was used for the tissue group). For tissues with limited sample availability, such as heart and kidney, a column-based isolation method using QIAmp Fast DNA Tissue Kit (Cat# 51404, Qiagen, Hilden, Germany) was performed. The remaining tissues, including stomach and lung samples, were processed using a magnetic bead-based extraction method with the MagnifiQ^TM^ 16 Genomic DNA instant kit (Cat# 604A-16U-256, A&A Biotechnology, Gdynia, Poland). DNA isolated with those methods yielded similar results in PCR, as established by initial experiments.

#### 2.4.1. Column-Based DNA Isolation

Small heart and kidney tissue fragments were bead-homogenized in AVE Buffer from the QIAmp Fast DNA Tissue Kit supplemented with VXL, DX Reagent, Proteinase K and RNase A, according to the protocol. Homogenization was performed using a TissueLyser II (Qiagen, Hilden, Germany) at 24 Hz for 30 s with a metal bead. Heart samples: the samples were incubated in Thermo Shaker TCS10 (Hangzhou Rui Cheng Instrument Co., Ltd., Hangzhou, China) for 20 min at 56 °C and 1000 rpm. Subsequently, the samples were subjected to a second homogenization step under the same conditions, followed by further incubation at 56 °C and 1000 rpm for 20 min or until no tissue fragment was visible. Kidney samples: after homogenization using TissueLyser II, the samples were incubated in a thermomixer for 15 min and 56 °C and 1000 rpm. Following lysis as outlined above, both heart and kidney samples were processed per manufacturer protocol. Briefly, clear lysates were mixed with Buffer MVL, transferred to the supplied spin columns and centrifuged at 14,000× *g* for 1 min, RT. Next, columns were washed with AW1 Buffer, centrifuged at 14,000× *g* for 1 min, RT, and then wash step was repeated with AW2 Buffer. After centrifugation, collection tube was changed, and empty columns were spined at 14,000× *g* for 2 min, RT. DNA was eluted using Buffer ATE. 25 µL of Buffer ATE was added to each column and incubated for 5 min at room temperature, followed by centrifugation at 14,000× *g* for 1 min, RT. The elution step was repeated with an additional 25 μL of ATE buffer under identical conditions. The collected eluates were subsequently analyzed for DNA concentration with Qubit 4 Fluorometer (Invitrogen, Carlsbad, CA, USA) using 1× dsDNA Broad Range Assay Kit (Cat# Q33266, Invitrogen, Carlsbad, CA, USA), and for purity using µDrop Plate (ThermoFisher Scientific, Waltham, MA, USA) and VarioScan Lux (ThermoFisher Scientific, Waltham, MA, USA).

#### 2.4.2. Magnetic Bead-Based DNA Isolation

Small tissue fragments were bead-homogenized in LSDE Buffer from MagnifiQ^TM^ 16 Genomic DNA instant kit, supplemented with 5 μL of 20 mg/mL RNase A (Cat# 12091-021, Invitrogen, Carlsbad, CA, USA). Homogenization was performed using a TissueLyser II (Qiagen, Hilden, Germany) at 24 Hz for 30 s, with metal beads. After the homogenization step, 40 μL of Proteinase K (Cat# K-PRK-12, A&A Biotechnology, Gdynia, Poland) was added, as per manufacturer’s instruction. Subsequently, the samples were incubated in Thermo Shaker TCS10 for 15 min at 50 °C and 1000 rpm. The samples were then subjected to a second homogenization step under the same conditions, followed by further incubation for 20 min at 50 °C and 1000 rpm, or until no tissue fragment was visible. Following lysis, the samples were centrifuged for 2 min 9000× *g*, and clear lysates were applied on XP-GD prefilled plate from the MagnifiQ^TM^ 16 Genomic DNA instant kit, as per manufacturer instruction. Plates containing the samples were processed using the Auto-Pure 32A (A&A Biotechnology, Gdynia, Poland) automated extraction system, using the dedicated DNA isolation protocol provided by the manufacturer. Next, samples were transferred into 1.5 mL clean Eppendorf tubes and incubated on a DynaMag^TM^-2 (Invitrogen, Carlsbad, CA, USA) magnetic stand for 5 min for additional removal of residual magnetic beads. The cleared samples were transferred to new tubes and analyzed for DNA concentration and purity as described above.

### 2.5. AAV Detection

Detection of wild type AAVs in human tissues was carried out through PCRs on gDNA isolated as described above. Q5 Hot Start High-Fidelity DNA Polymerase (Cat# M0493L, New England Biolabs, Ipswich, MA, USA) was used as per manufacturer instructions. For 15 μL total reaction volume 90 ng of gDNA was added for each reaction. Complete capsid sequence obtaining was aimed. Thus AAV genome rep and ITR sequences flanking cap (2660 bp) were targeted by primers Forward VG1202 (rep) and Reverse VG1203/4 (ITR), also paired with primers 18aS and 19S (targeting mid of cap) published previously [17] to obtain 255 bp-overlapping fragments (1215 bp and 1720 bp, respectively) in separate PCR reactions. Details of PCR targets and primers specificity are provided in supplemental materials (Figure S1, Table S1). All samples were screened for AAV cap sequence through at least two separate PCR runs targeting shorter fragment (18aS reverse primer) and additional PCRs for longer fragment (19S forward primer) and complete capsid sequence (VG1202 forward primer and VG1203/4 reverse) were performed. Two technical repeats were applied for each PCR round. For VG1202-18aS and 19S-VG1203/4 PCR conditions were as follows: denaturation step at 98 °C for 30 s, followed by 5 amplification cycles: 98 °C for 10 s, 67 °C annealing for 30 s, 72 °C for 90 s, then similar 33 cycles with annealing in 69 °C and final extension 72 °C for 2 min. For VG1202-VG1203/4 primer pair conditions were as follows: 98 °C for 30 s, then 39 cycles of 98 °C for 10 s, 72 °C for 2 min 20 s finalized with 72 °C for 2 min. PCR products were visualized by electrophoresis on 1% agarose gel. Samples that were positive in at least two separate PCR reactions were considered positive for the presence of AAV.

### 2.6. ELISA for Anti-AAV2 Antibodies

Anti-AAV2 IgG antibodies in serum were detected by ELISA (Enzyme-Linked Immunosorbent Assay). Serum samples were thawed on ice, centrifuged at 2000× *g* for 10 min at 4 °C before testing. ELISA Plate-coating Buffer (1×), Reagent Diluent Concentrate 2 (10×), Wash Buffer (25×), TMB ELISA Substrate (1×), and Stop Solution (1×) were supplied with DuoSet ELISA Ancillary Reagent Kit2 (Cat# DY008B, R&D Systems, Minneapolis, MN, USA). AAV2 vector solution containing 13 × 10^9^ vg per mL of Plate-coating Buffer was used to coat 384-wells plates (25 µL per well), at 4 °C, overnight. Following coating, the wells were washed 3 times with 1× Wash Buffer, 80 µL per wash and blocked with 75 μL of 1× Reagent Diluent for 2 h at RT. Subsequently, 25 µL of serum diluted 1:100 in 1× Reagent Diluent was added per well and incubated for 2 h at 4 °C (optimal serum dilution was established in preliminary experiments). After the incubation with serum, wells were washed 3 times (as previously) and incubated with goat anti-human IgG secondary antibody conjugated with HRP (Cat# 31413, lot# UG2813252, Invitrogen, Carlsbad, CA, USA, dilution 1:2000 in 1× Reagent Diluent) for 1 h at RT. Then, 5 washes were performed and 25 μL of TMB Substrate was added for 10 min. The enzymatic reaction was inactivated by adding 12.5 μL of Stop Solution. Optical density (OD) (at 450 nm) was measured using plate spectrophotometric reader (VarioScan Lux, ThermoFisher Scientific, Waltham, MA, USA). The CUT-OFF was set at OD = 0.25. Samples were considered negative if OD < CUT-OFF; Samples were considered as a positive if OD ≥ CUT-OFF. ELISA was performed in technical duplicates within a single independent experiment. To confirm validity of ELISA, we used IVIG and Positive control from commercial anti-AAV2 IgG ELISA (Abbexa cat no abx050304) which yielded positive signal in 1:100 dilution, implying non inferior sensitivity of the in-house ELISA. IVIG positive signal was detected up to 1:1000 dilution.

### 2.7. AAV Production

Human Embryonic Kidney 293T (HEK293T) cells were obtained from Cell Lines Services GmbH (Cat# 300189, CLS, Eppelheim, Germany). Recombinant AAV capsids were produced in adherent HEK293T cells using standard triple transfection protocol [18]. Briefly, cells were seeded onto 15 cm culture dishes at a seeding density 12.5 × 10^6^ cells per dish in Dulbecco’s Modified Eagle Medium High Glucose (4.5 g/L glucose) (Cat# 11965-02, Gibco, Paisley, UK) supplemented with 10% Fetal Bovine Serum (Cat# 12006C, Sigma-Aldrich, Barueri, Brazil) and 1× Penicillin-Streptomycin (P/S) solution (Cat# P4333, Sigma-Aldrich, Rehovot, Israel) and cultured at 37 °C in a humidified atmosphere containing 5% CO_2_. The next day, once the cells reached 70–90% confluency, the culture medium was replaced with 15 mL of fresh media supplemented with 25 mM HEPES (Cat# 15630-080, Gibco, Taipei City, Taiwan). After 2 h cells were transfected using the PEI (polyethylenimine) (Cat# 49553-93-7, Polysciences, Warrington, PA, USA) at PEI:DNA ratio 2:1. The plasmid mixture contained the AAV transfer plasmid encoding CMV-GFP reporter cassette (pTransfer), the RepCap helper plasmid (pHelper) and the adenoviral helper plasmid (pAd5) at 1:1:2 molar ratio. Mix contained DNA and PEI complexes was formed in OptiMEM (Cat# 31985, Gibco, Paisley, UK) medium, incubated for 10 min at room temperature, and added dropwise to the cells. Transfected cells were maintained for 72 h under standard culture conditions.

At 72 h post-transfection, cells and culture medium were harvested. Cell were pelleted by centrifugation (5250× *g*, 15 min, 4 °C) and cell pellet was resuspended in 10 mM Tris-MgCl_2_ buffer (pH 8.5), and subjected to three freeze (dry ice + 70% ethanol)/thaw (in 37 °C water bath) cycles to break the cells and release intracellular viral particles.

In parallel, 40% PEG/2.5 M NaCl solution (prepared by dissolving PEG 8000 (Cat# 0159-2.5, VWR, Radnor, PA, USA) in 5 M NaCl (Cat# S6546, Sigma-Aldrich, St. Louis, MO, USA)) was added to the cell-free culture supernatant to the final concentrations of 8% PEG and 0.5 M NaCl. Preparation was incubated overnight at 4 °C to precipitate extracellular viral particles.

Following centrifugation (5250× *g*, 15 min, 4 °C), the PEG8000 pellet containing AAV particles was resuspended in 10 mM Tris-MgCl_2_ buffer (pH 8.0) and mixed with the lysed cell fraction.

The total lysate was subjected to digestion with 200 U/mL Benzonase (Cat# 1.01695.0002, Merck, Søborg, Denmark) at 37 °C for 1.5 h. Next, 1.25 mL 5 M NaCl was added and 1:20 volume of 10% DOC (sodium deoxycholate) (Cat# D6750, Sigma-Aldrich, Auckland, New Zealand). After incubation for 30 min at 37 °C, the supernatant containing AAV was separated from the precipitate using centrifugation at 5250× *g* at 4 °C for 30 min.

AAV vectors were purified using an iodixanol-based (OptiPrep Density Gradient Medium, Cat# D1556, Sigma-Aldrich, Darmstadt, Germany) density gradient ultracentrifugation method as reported [19]. Briefly, the iodixanol gradients consisting of 15%, 25%, 40%, and 60% layers was formed and the lysate was loaded as the top layer in OptiSeal tubes (Cat# 361625, Beckman Coulter, Brea, CA, USA) and subsequently centrifuged in a Beckman Coulter Optima XPN Ultracentrifuge using 70Ti rotor at 58,000 rpm for 2 h and 10 min at 18 °C. After centrifuging, AAV containing 40% fraction was removed and passed through a 0.22 µm syringe filter.

The iodixanol fraction containing the vector was subjected to 5 cycles of buffer-exchange using UV sterilized 100 kDa pre-wet Amicon Ultra Centrifugal Units (Cat# UFC9010, Merck, Arklow, Ireland) and PBS containing 0.001% Pluronic F68 (Cat# 24040-032, Gibco, Waltham, MA, USA). Following buffer exchange, the sample was concentrated to 200 µL using the same Amicon filters. The final AAV vector stocks were collected into sterile, endotoxin-free 1.5 mL Eppendorf tubes for storage at 4 °C until use.

The DNA genome titer of the purified AAV vector stocks was measured by dPCR on the QIAcuity Digital PCR System (Qiagen, Hilden, Germany) using QIAcuity EvaGreen Mastermix (Cat# 250111, Qiagen, Hilden, Germany) with 0.4 μM eGFP primers (F: 5′-TCAAGATCCGCCACAACATC-3′; R: 5′-TTCTCGTTGGGGTCTTTGCT-3′) as previously described [20] and dPCR program: denaturation step at 95 °C for 2 min, followed by 40 amplification cycles: 95 °C for 15 s, 60 °C for 15 s, 72 °C for 15 s, and cooling down at 40 °C for 5 min.

### 2.8. Neutralization Assay

To analyze reactivity of anti-AAV NAbs, a neutralization assay on HEK293T cells was performed. Cells were seeded in 96-well plates using standard DMEM media as outlined above. The cells were allowed to reach 80% confluency before being transduced with AAV2-CAG-GFP reporter vector +/− human sera.

Serum samples were diluted 5× (optimal serum dilution was established in preliminary experiments) in culture medium and highly purified AAV2-CAG-GFP reporter vector (MOI = 5 × 10^4^) was added at 1:1 ratio and incubated for 2 h at 37 °C (final serum dilution 10×). Subsequently, the vector-serum mixture was added to the cells. Vector incubated with same volume of DMEM was used as control. After 24 h, GFP transgene expression was quantified by fluorescence measurement on a plate reader (VarioScan Lux, ThermoFisher Scientific, Waltham, MA, USA). Results were normalized to the level of GFP in control HEK293T cells transduced with AAV2-CAG-GFP not treated with serum (assigned value 100%). The neutralization assay was performed in technical duplicate within a single independent experiment using a single-point dilution approach.

### 2.9. Statistical Analysis

Frequency of AAV DNA-positive samples from patients was statistically tested with two-step approach based on contingency tables analysis. Initially, chi-squared value was calculated for testing whether AAVs presence differed between groups (tissue types). For cardiac tissues an Exact Fisher test was applied, accordingly. Subsequently, multiple (binary) logistic regression was applied for AAV prevalence (variable AAV^pos^/AAV^neg^) with four attributed Tissue type levels: 1—Cardiac (Atrium or Vessel), 2—Stomach, 3—Kidney, 4—Lung in order to support fitness of model (AAV^pos^/AAV^neg^ Tissue type) and whether differences between each group were significant.

The seroprevalence was calculated as the percentage of serum samples positive for anti-AAV2 IgGs (with OD > 0.25 in 1:100 serum dilution) in the total number of available samples and in each patient/tissue group.

To assess the differences in distribution of positive and negative samples, one sample Wilcoxon test (with proportion tested 1:1) or a chi-square test were used. For transduction experiments, results were analyzed with one sample Wilcoxon test by comparing transduction percentages to a theoretical median of 100% (no change in transduction compared to untreated AAV2). To analyze differences between groups, Mann–Whitney or Kruskal–Wallis tests with multiple comparison correction were applied. For correlations, Spearman analysis was performed. All statistical analyses were performed with Graph Pad Prism v.11. Results were considered statistically significant when *p* value ≤ 0.05 and are denoted on the graphs with asterisk.

## 3. Results

### 3.1. AAV Status of Studied Samples

**Figure 1:**
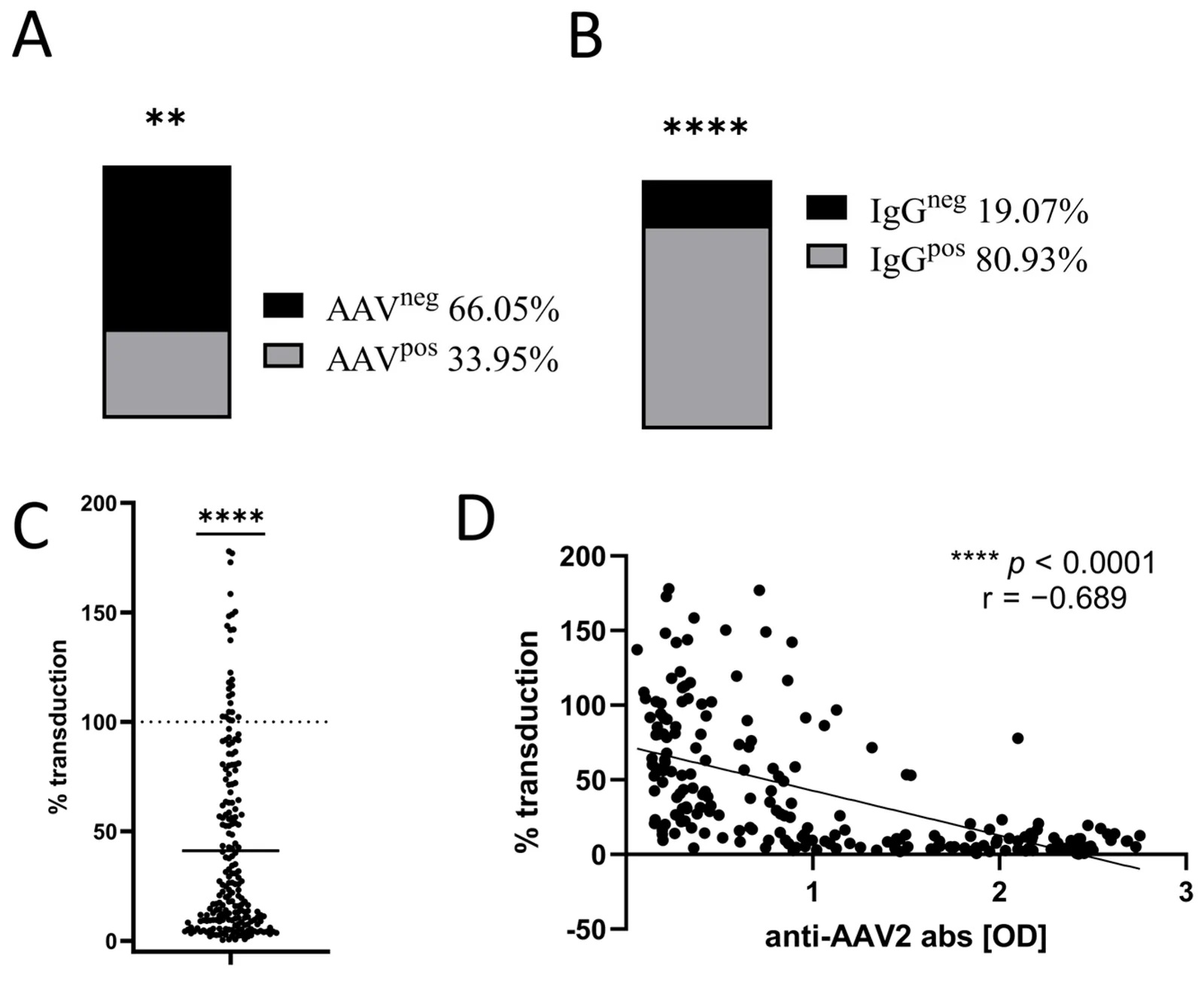
Frequency of AAV capsid sequences and anti-AAV2 antibodies in the entire study cohort. (**A**) Percentage of tissue samples positive for AAV capsid sequence, *n* = 215, ** *p* < 0.01 in chi-square test (compared to 1:1 ratio) (**B**) Percentage of AAV2 seropositive sera, *n* = 215, **** *p* < 0.0001 in chi-square test (compared to 1:1 ratio). (**C**) Change in the efficiency of AAV2 in vitro transduction following incubations with patient sera. Values are presented as percentages compared to control cells (assigned value 100% as indicated by the dotted line), *n* = 209, **** *p* < 0.001 in Wilcoxon Signed Rank Test (**D**) Correlation between anti-AAV2 antibodies [OD] and % change in in vitro transduction level, *n* = 209, Spearman r = −0.689, **** *p* < 0.0001.

We recruited 215 patients for tissue sample collection, including 99 patients from which we harvested fragments of stomach tissues, 46 patients from which kidney biopsies were obtained, 24 lung biopsy donors, 14 heart atrium biopsies, five aorta biopsies, as well as 27 patients from which we obtained both atrium and aorta samples. Each tissue sample was assayed for the presence of endogenous AAV in genomic DNA (gDNA) by PCR targeting AAV capsid gene flanking regions, as described in the Materials and Methods Section. Samples were scored as positive for AAV presence if any of the targeted capsid regions were detected at least twice out of four individual PCR reactions. In total, we detected AAV in 51 out of 215 samples (33.95%) (Figure 1A).

From each of the 215 patients, serum was also obtained and assayed by ELISA for the presence of anti-AAV2 specific IgG antibodies. Samples were considered positive if serum dilution 1:100 (optimal dilution determined in preliminary results) had OD > 0.25. In total, 80.93% of patients (174 out of 215) tested seropositive for anti-AAV2 IgG (Figure 1B).

Serum samples from patients in the study were also used to perform an AAV neutralization assay. Results were normalized to the level of GFP in control HEK293T cells transduced with AAV2-CAG-GFP not treated with serum (assigned value 100%). Figure 1C shows the GFP levels for all samples.

Most of the patient sera samples tested significantly reduced the level of transduction to mean level of 40% (termed fold change % transduction on graphs, where 100% was the GFP level in cells transduced with control AAV2). Some of the samples tested (16%) reduced the transduction to undetectable levels, while some sera (*n* = 26, 12%) led to an increased levels of transduction as compared to the control group.

Negative correlation between the levels of anti-AAV2 antibodies (as detected by ELISA) and the results of the in vitro transduction assay (Spearman r = −0.689, *p* < 0.0001, Figure 1D) suggested that the anti-AAV antibodies detected contained anti-AAV2 neutralizing antibodies (NAbs).

### 3.2. AAV Capsid Sequences Are Present in Different Tissues with Similar Prevalence

**Figure 2:**
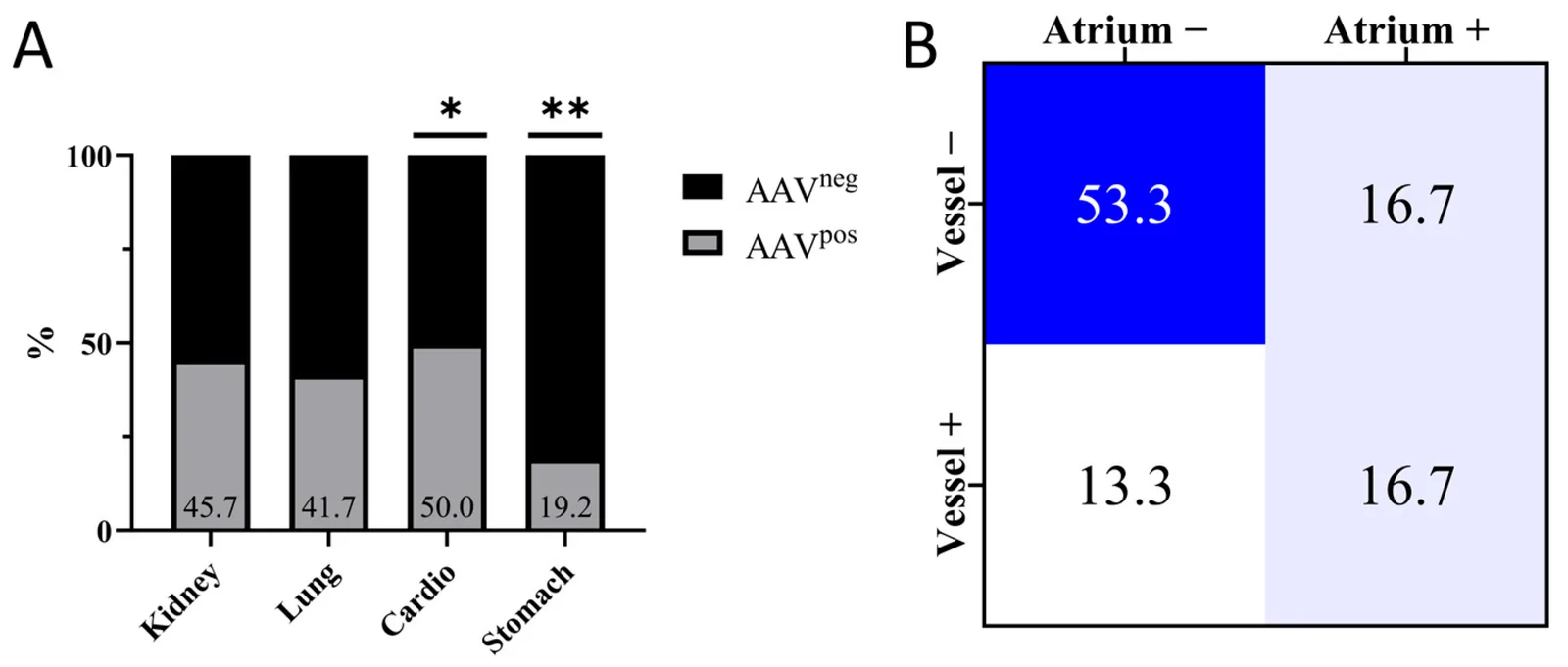
Distribution of AAV capsid sequences detected by PCR in different patient groups. (**A**) Percentage of positive samples per tissue group, *n* = 215, * *p* < 0.05, ** *p* < 0.01 in chi-square test (compared to whole cohort prevalence). (**B**) Cross-tabulation of AAV capsid sequence detection (% of total 30 samples) between atrium and vessel samples in patients, for whom both tissues were obtained.

The prevalence of AAV capsid sequences ranged from 19 to 50% in individual tissue groups, with cardio (50%) and stomach (19.2%) groups having the frequency of AAV positivity significantly different from the whole cohort (33.95%). The stomach group had the lowest percentage of positive samples, while cardio tissues had the highest frequency of positivity (Figure 2A). Among patients from the cardio group, from which both atrium and vessel were obtained (*n* = 30), AAV detection rates were 53.33% in atrium and 30% in vessel samples, respectively. However, only 16.7% of patients were ‘double positive’ with AAV detected in both the atrium and vessel tissues (Figure 2B).

### 3.3. Distribution of Anti-AAV2 Antibodies in Different Groups

The availability of DNA from different tissues and access to corresponding serum samples allowed a cross-analysis of studied groups depending on the AAV status of the tissue and/or sera.

**Figure 3:**
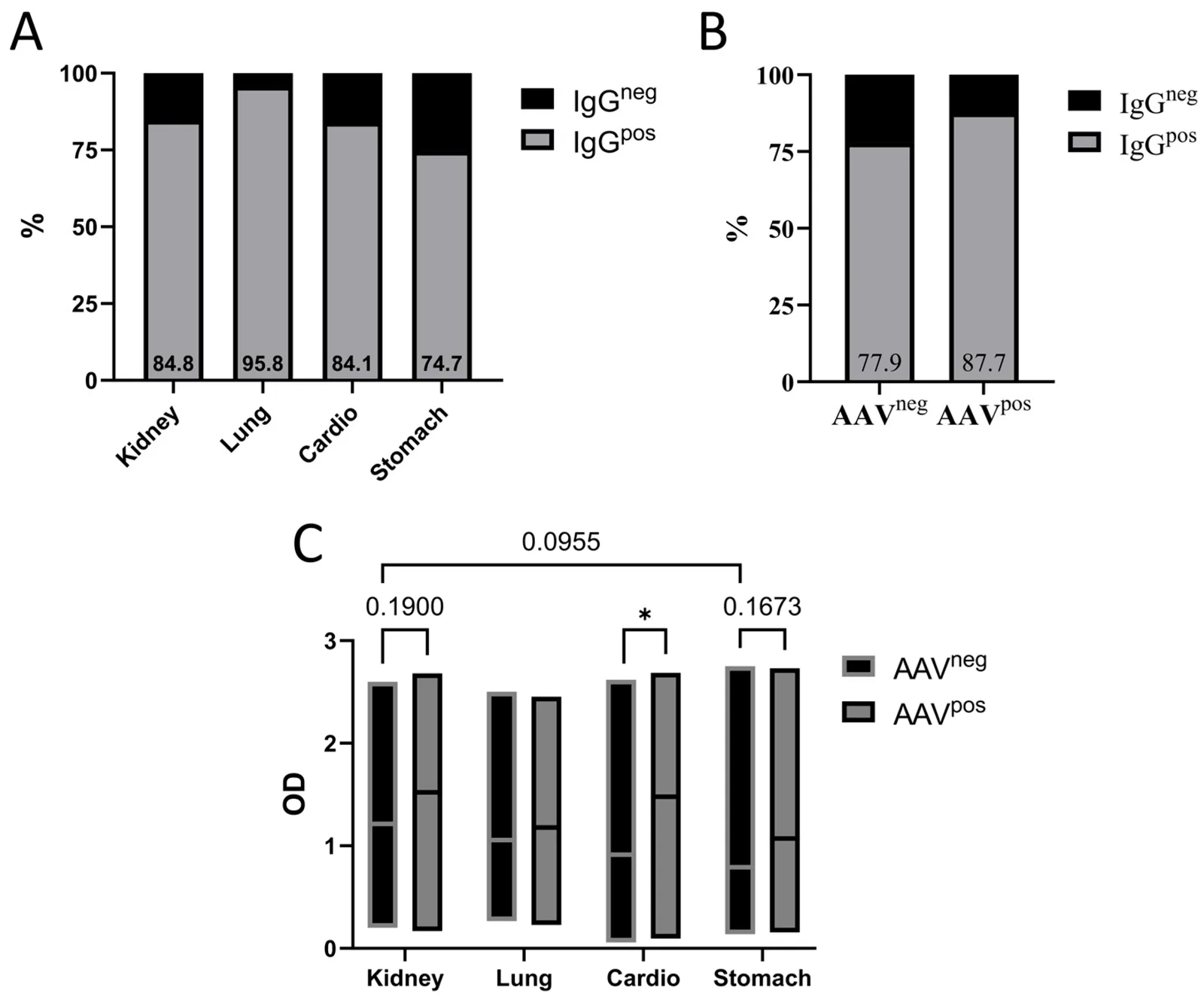
Anti-AAV2 seropositivity tested in serum samples. (**A**) Percentage of seropositive samples according to tissue group, *n* = 213. None of the groups showed significant difference compared to whole cohort prevalence (81%). (**B**) Percentage of seropositive samples in the entire cohort, divided into groups depending on the AAV sequence presence: AAV-negative (AAV^neg^) and AAV-positive (AAV^pos^), *n* = 213. (**C**) Level of anti-AAV2 antibodies (mean OD values) in tissue groups depending on AAV sequence presence, *n* = 213 * *p* < 0.05 in 2way ANOVA with Tukey’s multiple comparisons test.

When analyzed separately by tissue group, seropositivity ranged from 74% to 95%. Except for the lung group, these values did not differ significantly from the prevalence in the overall cohort (chi-square test compared with the total frequency) (Figure 3A). In the lung group only 1 sample (out of *n* = 24 collected lung samples) was negative for anti-AAV2 IgG. When the seropositivity was correlated to the presence of AAV genomes in corresponding gDNA samples, not surprisingly, it was higher in patients with AAV detected in tissue samples than in those without detected AAV (*p* value = 0.0821 in Chi-square test, Figure 3B), even though one could expect the difference in seropositivity to be more significant between these two groups. When each tissue group was considered separately, the strongest correlation between the AAV genome and anti-AAV2 antibodies presence was observed in the cardio group (Figure 3C).

Detailed correlation analysis among patients for whom both atrium and aorta samples were analyzed for AAV genomes, seropositivity was lowest in the double-negative group (66.7%) and reached 100% in the double-positive group. Interestingly, also all patients who were AAV-positive only in the atrium were seropositive. However, this finding may reflect the small sample size in this group (*n* = 5; Figure S2).

### 3.4. Neutralizing Potential of Patient Sera

**Figure 4:**
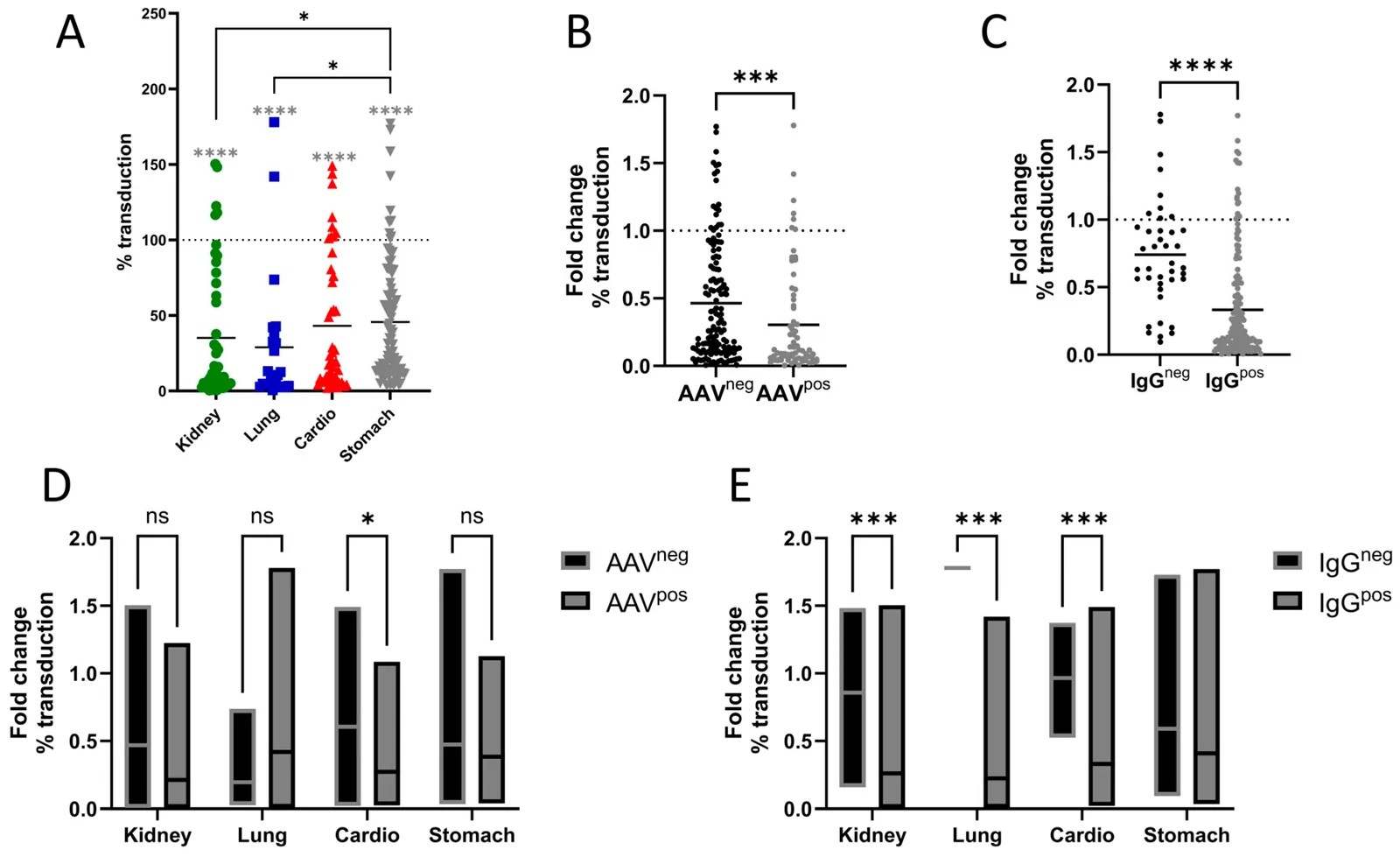
Comparison of transduction efficiency depending on AAV status in tissue or serum. (**A**) Change in transduction level according to tissue type, *n* = 207, black * *p* < 0.05 in Kruskal–Wallis test, gray **** in one sample Wilcoxon test comparing to a theoretical median of 100%. (**B**) Change in transduction level according to AAV sequence presence, *n* = 207, *** *p* < 0.001 in Mann–Whitney test. (**C**) Change in transduction level according to seropositivity, *n* = 209, **** *p* < 0.0001 in Mann–Whitney test. (**D**) Change in transduction level according to tissue type and classified as AAV sequence-negative or AAV sequence-positive, *n* = 207, * *p* < 0.05 in 2way ANOVA with Šídák’s multiple comparisons test. (**E**) Percentage of transduced cells in the entire study cohort, divided into groups based on anti-AAV2 seropositivity, *n* = 207, *** *p* < 0.001 in 2way ANOVA with Šídák’s multiple comparisons test.

We next wanted to evaluate the ability of anti-AAV Nabs to neutralize AAV function. To do so, we performed an in vitro AAV neutralization assay using reported AAV2-CAG-GFP vector. The transduction was reduced to a mean values ranging from 26 to 40% between tissue groups, with lung group showing the strongest effect (reduced to 26% compared to untreated AAVs). However, the mean neutralization was not significantly different between groups (Figure 4A).

Next, we correlated the levels of neutralization of AAV transduction with the presence of AAV genome in tissues and the presence of anti-AAV2 antibodies as detected by ELISA. Serum from patients with detectable AAV genomes showed significantly stronger levels of AAV neutralization than serum from ‘AAV negative patients’ (Figure 4B). However, when the tissue groups were analyzed separately, this difference remained statistically significant only in the cardiovascular group, where AAV-positive patients showed stronger neutralizing activity than AAV-negative patients (Figure 4D). Interestingly, the lung group showed the opposite trend, with lower neutralizing activity in the AAV-positive group, although this difference was not statistically significant (means % of transduction AAV^neg^ = 19.6%, AAV^pos^ = 41.9%, *p* = 0.7952). Among the cardiovascular groups for which both atrium and aorta samples were available, the strongest reduction in AAV transduction was observed in patients who were AAV-positive in both tissues. In contrast, sera from double-negative patients showed the highest transduction levels and therefore the lowest neutralizing activity, although transduction remained lower than the control (100%) (Figure S2).

As expected, seropositive samples showed stronger neutralization (Figure 4C) and this effect was observed in all tissue groups apart from stomach (Figure 4E).

## 4. Discussion

In the studied cohort, anti-AAV2 antibody prevalence was 80.9%, which is relatively high when compared to published results for other populations [12,21,22,23,24]. This result is not totally unexpected, however, as global prevalence of preexisting immunity to different AAV serotypes is known to be variable and dependent on many factors, including geographical localization, age, gender, and socioeconomic status [12,21,22,23,24]. Seroprevalence ranging from 22% up to 97% was recently reported [12,21,22,23,24]; therefore, our cohort, although falling within the higher end of this spectrum, is not outside the previously published range. High percentage of positive samples can be partially explained by the characteristics of patients enrolled in our study. All patients were adults, and age has been shown to correlate positively with the prevalence of anti-AAV NAbs. Furthermore, the donors were patients who were enrolled to the hospital for planned surgeries, mostly due to chronic diseases, suggesting that many of them had undergo prior hospitalization(s). As it had been shown before that hospitalization and surgery correlate with increased prevalence of anti-AAV antibodies [25], this could, at least partially, explain the high level of seropositivity among our study donors. High seroprevalence in Polish population align with findings reported by Greenberg et al., showing that the prevalence of AAV1 NAbs levels were the highest in Poland and Hungary (79% in both) compared to other tested countries [26].

Serum samples from seropositive donors had significantly stronger neutralizing potential as evident by AAV transduction inhibition assay and the level of transduction inhibition correlated with the titer of anti-AAV antibodies. Previous studies have established that anti-AAV antibodies are an important component of the humoral immune response to AAV capsids [27]. However, humoral and cellular immune responses to AAV capsids do not necessarily correlate. In particular, Kruzik et al. reported no correlation between anti-AAV antibody responses and anti-AAV T-cell responses, and neutralizing antibody titers did not significantly correlate with anti-AAV T-cell responses [28]. Thus, while anti-AAV antibody levels provide information about pre-existing humoral immunity, they should not be considered a reliable biomarker for predicting capsid-specific cellular or cytotoxic lymphocyte responses or the overall anti-vector immune response following AAV administration. Importantly, patient serum is a source of not only neutralizing antibodies but also of a plethora of factors that can improve capsid performance, potentially via a non-antibody related mechanism. Indeed, in our study we observed that at the dilution used, some sera samples improved, rather than decreased, in vitro transduction. Similar observations have been previously reported by others [29], suggesting that some components present in the serum matrix (e.g., growth factors) can affect capsid transduction effectiveness, either inhibiting or enhancing it, both in vitro and potentially in vivo [30].

Regardless of the specific reasons for such high prevalence of pre-existing anti-AAV antibodies among the adult Polish patients enrolled in this study, it should be noted that considering the fact that pre-existence of anti-AAV antibodies is a frequent exclusion criterion in AAV-based clinical studies, and data presented in this study could imply potential barriers into the enrolment of polish adult patients into AAV-based clinical studies.

The data on natural AAV genomes detection in human tissue is sparse. In our study it ranged from 19 to 50% depending on tissues type, which is similar to other reports [12,24]. It has been previously reported that wild type (non-vector) AAV genomic DNA in latent infection may be integrated into host genome, with affinity to human chromosome 19 (q13.42) locus, known as the AAVS1 [31,32,33,34]. However, during natural infection, AAV genome can also integrate semi-randomly throughout the host genome, a process that often leads to the AAVs genome being fragmented and/or rearranged [35,36,37,38,39,40]. This contributes to frequent difficulty in recovering the complete AAV genomes from host samples [41,42]. Furthermore, in some cases, established PCR-based methods allow one to recover fragment of the *cap* gene, but it proves very difficult, or impossible, to amplify the remaining sequences to complete the AAV *cap* gene. This might be explained by fragmentation of AAV genome and/or absence of specific sequences targeted by AAV recovery primers, despite the fact that certain level of sequence heterogeneity is usually built into the design of degenerate recovery primers. When analyzing the human tissue samples for the presence of AAV genome, we experienced similar difficulties—for some of the gDNA samples we were able to obtain an amplicon of the AAV capsid-coding region only once, despite multiple attempts by different operators. In such cases, the single positive signal was classified as “false positive” and the samples were considered negative for the presence of AAV genome. For that reason, the frequency of AAV in patient samples reported here is very conservative and probably underestimated, and thus treated as the minimum infection rate.

Despite the outlined issues that prevented us from amplifying the full capsid sequences from proportion of tissue samples, the still relatively high rate of AAV prevalence and tissue distribution positions human tissues as a potentially rich source of AAV variants. Natural AAV capsid variants recovered from human tissues could either directly have utility in preclinical or clinical applications, or could serve as starting scaffolds for further capsid bioengineering. This is especially so, as there is evidence that natural AAVs may maintain tissue specificity of the organ from which they were isolated [43,44]. Thus AAV isolated from individual clinically relevant tissues, such as lung or cardiac tissues, could enable more precise tissue-specific delivery, especially as most of therapeutic vectors are sequestered in the liver and targeting other organs is limited [45].

Natural AAVs have evolved to enter cells through interactions with specific glycans (mainly sialic acid, galactose, and heparan sulfate) and receptors present on target cells [46]. Therefore, studies of novel AAV variants isolated from specific clinically relevant tissue targets may lead to the identification of specific tissue-restrictive receptor targets, which could subsequently provide us with novel basis for vector bioengineering [47].

Our data show that both seropositivity and neutralizing activity were higher in AAV-PCR positive patients. This is not surprising, since positive AAV PCR indicates that at the time of tissue harvest (1) the subject had an ongoing AAV infection; (2) the subject was previously exposed to AAV, and the viral genome was present is the tissues as integrated or non-integrated episomes; or (3) that the subject had an AAV pro-vector in the genome as a consequence of germline infection and genomic integration that could have occurred many generations earlier, and potential scenarios 1 and 2 would lead to immune system activation and seropositivity. Furthermore, there is additional important difference between testing seropositive for AAV and having detectable AAV in tissue samples. Systemic exposure to AAV would lead to the activation of the immune system, and thus seropositivity; however, during an AAV infection, AAV does not have to integrate into host genomic DNA and if such integration does occur, it can be rare and restricted to specific tissue(s). During replication, AAV evolve fast [20,42,48]; therefore, during active infection multiple variants are present in the infected subject, with individual AAV variants potentially having unique affinity for various tissues and cell types. Thus, negative tissue sample for the presence of AAV genome does not necessarily indicate that the subject was not exposed to natural AAV.

In our cohort, we identified eight patients that had AAV detected in at least one tissue, yet anti-AAV2 antibodies could not be detected by ELISA. Additionally, most of them (*n* = 5) also did not significantly alter in vitro transduction in the AAV neutralization assay. They were distributed randomly in the tissue groups and while those subjects could have inherited AAV in their genome (as per scenario 3 outlined above) it is also possible that they were exposed to an AAV virus, but the exposure did not activate their immune system. It is also possible that capsids identified in those sample may have some specific immunological properties making them “invisible” to the immune system; however, further studies are required to explore this hypothesis.

Of note are the results obtained from cardio group where both atrium and vessel could be assayed. Although total AAV prevalence in this group was the highest (50%), only 29% patients were “double positive” by PCR. It may be that AAVs reside in specific organs rather than are present systemically (as discussed above), which is aligned with the notion of AAV organo-specificity. Although, we could not test additional patients or additional tissue types from seropositive patients, bio-distribution of capsid DNA and corresponding seropositivity is an interesting direction of ongoing and future AAV biology research. Additionally, the cardio group was characterized by a higher prevalence of AAV sequences in tissue (50%) and those patients had particularly strong immune response to AAV2 (level of total antibodies and Nabs). The information about natural AAV infections in human is limited, therefore it is difficult to state why cardiac tissues had more AAVs. Stronger immune response may suggest that natural cardiac AAVs are more immunogenic, however we could not confirm this. In fact, Zeissler et al. shown that one of their natural AAV isolates from ventricular tissue, was characterized by low immunogenicity [49]. On the other hand, cardiac tissue or cardiosurgery patients specifically can be more exposed/sensitive to AAV infection. However, in a 2004 study it was shown that heart tissues had one of the lowest frequencies of AAV detection although the whole studied group had lower general AAV prevalence (19%), compared to our cohort [12].

The relatively high prevalence of AAV sequences in cardiovascular tissues in our study raises the question of whether tissue-specific expression of AAV attachment and entry factors could contribute to this observation. Prototypical AAV2 binds initially to cell-surface heparan sulfate proteoglycans (HSPGs), with key capsid residues including R585 and R588 contributing to this interaction [50,51]. However, it should be emphasized that the significance of this interaction for natural tissue tropism remains not fully understood. Cabanes-Creus et al. suggest that the AAV2-HSPG interaction may merely be an adaptation of HEK293 cells, frequently used in in vitro models [48]. Thus, the mechanism of AAV tissue tropism is multifactorial: FGFR1, αVβ5 integrin, and α5β1 integrin have historically been proposed as AAV2 co-receptors or entry-promoting factors [52,53,54]. More recently, AAVR/KIAA0319L was identified by unbiased genetic screening as a critical receptor for AAV2 infection, and AAVR loss strongly reduces AAV2 transduction across multiple mammalian cell types [55]. Available human tissue and single-cell atlases show that genes encoding these entry-associated factors are expressed across multiple organ and cell compartments rather than exhibiting a simple cardiac-specific distribution [56,57]. Therefore, receptor availability could contribute to AAV attachment or uptake in cardiovascular cells, but currently does not provide a sufficient explanation for the higher frequency of AAV DNA detected in cardiovascular specimens. Importantly, the endogenous AAV sequences detected in the present study were identified by cap-region PCR and should not be assumed to represent canonical laboratory AAV2 (present study). This distinction is biologically relevant because naturally occurring AAV2-like isolates recovered from human tissues can lack the canonical HSPG-binding phenotype, including changes affecting residues R585 and R588 [58], and adaptation to strong HSPG binding can alter in vivo tropism [48,59]. Finally, PCR detection reflects the presence or persistence of viral DNA rather than active viral entry; naturally acquired AAV genomes can persist in human tissues as extrachromosomal circular forms [11,60]. Consequently, tissue-specific persistence, prior exposure, helper-virus history, cellular composition and receptor accessibility, in addition to receptor expression, may contribute to the cardiovascular enrichment observed in this cohort.

Several limitations of our study should be noted. First, the cohort consisted only of hospitalized adult patients undergoing surgeries, and thus may not be a proper representation of the general Polish population. Second, in most cases, only a single tissue type was obtained from each patient, not allowing us to gain a better understanding of biodistribution of natural AAV variants in various tissues. This also does not fully characterize AAV presence in an individual patients, especially in respect to the presence of anti-AAV antibodies. Also, AAV detection by PCR does not specify the current status of infection, as this method cannot distinguish a latent AAV genome that is integrated into the host genome from genome purified from AAV particles present during an active ongoing infection. Finally, only anti-AAV2 antibody response was studied, while natural AAV cross- reactivity will affect also other serotypes and variants. Another methodological limitation is that the ELISA and neutralization assays were conducted using a single-point screening (100 times dilution) across a single independent experiment, rather than a full multi-point dilution series to determine ND_50_ titters. While our approach provided efficient primary screening, it represents a methodological limitation in capturing the quantitively titration curves and may not be directly comparable to other studies due to these methodological variables. In addition, while this study does not provide any insights into the pediatric population, considering the large number of gene therapies in development for pediatric indications, this study highlights the important need to conduct a serological analysis to assess the frequency of pre-existing anti-AAV antibodies among Polish pediatric cohort.

## 5. Conclusions

In conclusion, we showed that preexisting humoral immunity to AAVs is highly prevalent in the Polish population and that natural capsid sequences can be found in a substantial proportion of human samples. The imperfect overlap between seroprevalence, PCR positivity and neutralizing activity highlights the complexity of wild-type AAV infectivity in various tissues and activation of the immune system, but also points to the potential limitations to the application of AAV-based gene therapies in a wider population in Poland. At the same time, we show that human tissues serve as a rich reservoir of natural AAVs which can fuel efforts towards the development of novel vectors with clinically desirable properties.

## Data Availability

The data presented in this study are available upon request from the corresponding author.

## Supplementary Materials

The following supporting information can be downloaded at: https://www.mdpi.com/article/10.3390/v18101134/s1, Table S1: Nucleotide sequences of primers used for PCR reaction; Figure S1: AAV genome simple graph based on AAV2 genome (NC_001401.2) with mapped ITR, rep, cap, primer targets and amplicon size. Prepared with use of SnapGene 8.2.2 (Dotmatics) software; Figure S2: Immune responses in cardiovascular tissue patients, stratified into groups based on AAV PCR-positivity.

## Abbreviations

The following abbreviations are used in this manuscript:

AAV	Adeno-associated viruses
ELISA	Enzyme-linked immunosorbent assay
GFP	Green Fluorescent Protein
HEK293T	Human Embryonic Kidney Cell Line
ITR	Inverted Terminal Repeat
NAb	Neutralizing Antibodies
ORF	Open Reading Frames
PCR	Polymerase chain reaction
RT	Room temperature

## References

[1] Atchison R.W., Casto B.C., Hammon W.M. (1965). Adenovirus-Associated Defective Virus Particles. Science.

[2] Xu L., Yao S., Ding Y.E., Xie M., Feng D., Sha P., Tan L., Bei F., Yao Y. (2024). Designing and Optimizing AAV-Mediated Gene Therapy for Neurodegenerative Diseases: From Bench to Bedside. J. Transl. Med..

[3] Issa S.S., Shaimardanova A.A., Solovyeva V.V., Rizvanov A.A. (2023). Various AAV Serotypes and Their Applications in Gene Therapy: An Overview. Cells.

[4] Aksu Kuz C., Ning K., Hao S., Cheng F., Qiu J. (2024). Role of the Membrane-Associated Accessory Protein (MAAP) in Adeno-Associated Virus (AAV) Infection. J. Virol..

[5] di Minno M.N.D., Peyvandi F. (2026). Roctavian Withdrawal: Exploring the Gap between Innovation and Healthcare System Readiness in Hemophilia A Gene Therapy. Res. Pract. Thromb. Haemost..

[6] Park K.S., Cho Y.I., Mitragotri S., Zhao Z. (2026). Viral Vector-Based Gene Therapies in the Clinic: An Update. Bioeng. Transl. Med..

[7] Zhao Z., Anselmo A.C., Mitragotri S. (2022). Viral Vector-Based Gene Therapies in the Clinic. Bioeng. Transl. Med..

[8] Xue F., Ju M., Zhu T., Zhou Z., Sun J., Yang L., Yan Z., Zhou H., Du X., Zheng C. (2026). Factor IX-Padua AAV Gene Therapy in Hemophilia B: Phases 1/2 and 3 Trials. Nat. Med..

[9] Weinstein D.A., Derks T.G., Rodriguez-Buritica D.F., Ahmad A., Couce M.-L., Mitchell J.J., Riba-Wolman R., Mount M., Sallago J.B., Ross K.M. (2025). Safety and Efficacy of DTX401, an AAV8-Mediated Liver-Directed Gene Therapy, in Adults with Glycogen Storage Disease Type I a (GSDIa). J. Inherit. Metab. Dis..

[10] Wang J.-H., Gessler D.J., Zhan W., Gallagher T.L., Gao G. (2024). Adeno-Associated Virus as a Delivery Vector for Gene Therapy of Human Diseases. Signal Transduct. Target. Ther..

[11] Schnepp B.C., Jensen R.L., Chen C.-L., Johnson P.R., Clark K.R. (2005). Characterization of Adeno-Associated Virus Genomes Isolated from Human Tissues. J. Virol..

[12] Gao G., Vandenberghe L.H., Alvira M.R., Lu Y., Calcedo R., Zhou X., Wilson J.M. (2004). Clades of Adeno-Associated Viruses Are Widely Disseminated in Human Tissues. J. Virol..

[13] Boutin S., Monteilhet V., Veron P., Leborgne C., Benveniste O., Montus M.F., Masurier C. (2010). Prevalence of Serum IgG and Neutralizing Factors Against Adeno-Associated Virus (AAV) Types 1, 2, 5, 6, 8, and 9 in the Healthy Population: Implications for Gene Therapy Using AAV Vectors. Hum. Gene Ther..

[14] Louis Jeune V., Joergensen J.A., Hajjar R.J., Weber T. (2013). Pre-Existing Anti–Adeno-Associated Virus Antibodies as a Challenge in AAV Gene Therapy. Hum. Gene Ther. Methods.

[15] Calcedo R., Vandenberghe L.H., Gao G., Lin J., Wilson J.M. (2009). Worldwide Epidemiology of Neutralizing Antibodies to Adeno-Associated Viruses. J. Infect. Dis..

[16] Perocheau D.P., Cunningham S., Lee J., Antinao Diaz J., Waddington S.N., Gilmour K., Eaglestone S., Lisowski L., Thrasher A.J., Alexander I.E. (2019). Age-Related Seroprevalence of Antibodies Against AAV-LK03 in a UK Population Cohort. Hum. Gene Ther..

[17] Gao G.-P., Alvira M.R., Wang L., Calcedo R., Johnston J., Wilson J.M. (2002). Novel Adeno-Associated Viruses from Rhesus Monkeys as Vectors for Human Gene Therapy. Proc. Natl. Acad. Sci. USA.

[18] Westhaus A., Cabanes-Creus M., Rybicki A., Baltazar G., Navarro R.G., Zhu E., Drouyer M., Knight M., Albu R.F., Ng B.H. (2020). High-Throughput In Vitro, Ex Vivo, and In Vivo Screen of Adeno-Associated Virus Vectors Based on Physical and Functional Transduction. Hum. Gene Ther..

[19] Strobel B., Miller F.D., Rist W., Lamla T. (2015). Comparative Analysis of Cesium Chloride- and Iodixanol-Based Purification of Recombinant Adeno-Associated Viral Vectors for Preclinical Applications. Hum. Gene Ther. Methods.

[20] Cabanes-Creus M., Ginn S.L., Amaya A.K., Liao S.H.Y., Westhaus A., Hallwirth C.V., Wilmott P., Ward J., Dilworth K.L., Santilli G. (2019). Codon-Optimization of Wild-Type Adeno-Associated Virus Capsid Sequences Enhances DNA Family Shuffling While Conserving Functionality. Mol. Ther. Methods Clin. Dev..

[21] Qin X., Li H., Zhao H., Xiang K., Liu S., Lou R., Liu P., Dai Y., Wang C., Zhang S. (2024). Prevalence of Neutralizing Antibodies Against AAV Serotypes 2 and 9 in Healthy Participants from Multiple Centers Across China and Patients with DMD/BMD. Hum. Gene Ther..

[22] Navarro-Oliveros M., Vidaurrazaga A., Soares Guerra G., Castellana D., Embade N., Millet O., Marigorta U.M., Abrescia N.G.A. (2024). Seroprevalence of Adeno-Associated Virus Types 1, 2, 3, 4, 5, 6, 8, and 9 in a Basque Cohort of Healthy Donors. Sci. Rep..

[23] Kashiwakura Y., Baatartsogt N., Yamazaki S., Nagao A., Amano K., Suzuki N., Matsushita T., Sawada A., Higasa S., Yamasaki N. (2022). The Seroprevalence of Neutralizing Antibodies against the Adeno-Associated Virus Capsids in Japanese Hemophiliacs. Mol. Ther. Methods Clin. Dev..

[24] Purohit N., Jain A., Mathews V., Jayandharan G.R. (2019). Molecular Characterization of Novel Adeno-Associated Virus Variants Infecting Human Tissues. Virus Res..

[25] Sierra-Delgado J.A., Likhite S., Bautista P.K., Gómez-Ochoa S.A., Echeverría L.E., Guío E., Vargas C., Serrano N.C., Meyer K.C., Rincon M.Y. (2023). Prevalence of Neutralizing Antibodies against Adeno-Associated Virus Serotypes 1, 2, and 9 in Non-Injected Latin American Patients with Heart Failure—ANVIAS Study. Int. J. Mol. Sci..

[26] Greenberg B., Butler J., Felker G.M., Ponikowski P., Voors A.A., Pogoda J.M., Provost R., Guerrero J., Hajjar R.J., Zsebo K.M. (2016). Prevalence of AAV1 Neutralizing Antibodies and Consequences for a Clinical Trial of Gene Transfer for Advanced Heart Failure. Gene Ther..

[27] Odenwald C., März M., Adam S., Prestel K., Hupe L., Queitsch I., del Rio Martinez J., Jafarov A., Edinger Z., Nelson J. (2025). Comparative Analysis of Total Antibody Titer and Neutralizing Antibody Titer: Correlation and Implications for AAV-Based Gene Therapies. Cytotherapy.

[28] Kruzik A., Fetahagic D., Hartlieb B., Dorn S., Koppensteiner H., Horling F.M., Scheiflinger F., Reipert B.M., de la Rosa M. (2019). Prevalence of Anti-Adeno-Associated Virus Immune Responses in International Cohorts of Healthy Donors. Mol. Ther.-Methods Clin. Dev..

[29] Kovács B., Szabó V., Horváth D., Dobos A.B., Nagy Z.Z., Vanduffel W., Szemlaky Z., Szepesi Á., Ulbert I., Rózsa B. (2026). Overcoming Matrix Effects in AAV Neutralization Assays with a Constant Serum Concentration Approach. Gene Ther..

[30] Gorovits B., Azadeh M., Buchlis G., Harrison T., Havert M., Jawa V., Long B., McNally J., Milton M., Nelson R. (2021). Evaluation of the Humoral Response to Adeno-Associated Virus-Based Gene Therapy Modalities Using Total Antibody Assays. AAPS J..

[31] Kotin R.M., Siniscalco M., Samulski R.J., Zhu X.D., Hunter L., Laughlin C.A., McLaughlin S., Muzyczka N., Rocchi M., Berns K.I. (1990). Site-Specific Integration by Adeno-Associated Virus. Proc. Natl. Acad. Sci. USA.

[32] Kotin R.M., Linden R.M., Berns K.I. (1992). Characterization of a Preferred Site on Human Chromosome 19q for Integration of Adeno-associated Virus DNA by Non-homologous Recombination. EMBO J..

[33] Samulski R.J. (1993). Adeno-Associated Virus: Integration at a Specific Chromosomal Locus. Curr. Opin. Genet. Dev..

[34] Samulski R.J., Zhu X., Xiao X., Brook J.D., Housman D.E., Epstein N., Hunter L.A. (1991). Targeted Integration of Adeno-associated Virus (AAV) into Human Chromosome 19. EMBO J..

[35] Dyall J., Szabo P., Berns K.I. (1999). Adeno-Associated Virus (AAV) Site-Specific Integration: Formation of AAV– AAVS1 Junctions in an in vitro System. Proc. Natl. Acad. Sci. USA.

[36] Dutheil N., Shi F., Dupressoir T., Linden R.M. (2000). Adeno-Associated Virus Site-Specifically Integrates into a Muscle-Specific DNA Region. Proc. Natl. Acad. Sci. USA.

[37] Logan G.J., Dane A.P., Hallwirth C.V., Smyth C.M., Wilkie E.E., Amaya A.K., Zhu E., Khandekar N., Ginn S.L., Liao S.H.Y. (2017). Identification of Liver-Specific Enhancer–Promoter Activity in the 3′ Untranslated Region of the Wild-Type AAV2 Genome. Nat. Genet..

[38] Hüser D., Gogol-Döring A., Lutter T., Weger S., Winter K., Hammer E.-M., Cathomen T., Reinert K., Heilbronn R. (2010). Integration Preferences of Wildtype AAV-2 for Consensus Rep-Binding Sites at Numerous Loci in the Human Genome. PLoS Pathog..

[39] Lamartina S., Sporeno E., Fattori E., Toniatti C. (2000). Characteristics of the Adeno-Associated Virus Preintegration Site in Human Chromosome 19: Open Chromatin Conformation and Transcription-Competent Environment. J. Virol..

[40] Young S.M., McCarty D.M., Degtyareva N., Samulski R.J. (2000). Roles of Adeno-Associated Virus Rep Protein and Human Chromosome 19 in Site-Specific Recombination. J. Virol..

[41] Lochrie M.A., Tatsuno G.P., Arbetman A.E., Jones K., Pater C., Smith P.H., McDonnell J.W., Zhou S.-Z., Kachi S., Kachi M. (2006). Adeno-Associated Virus (AAV) Capsid Genes Isolated from Rat and Mouse Liver Genomic DNA Define Two New AAV Species Distantly Related to AAV-5. Virology.

[42] Gao G., Alvira M.R., Somanathan S., Lu Y., Vandenberghe L.H., Rux J.J., Calcedo R., Sanmiguel J., Abbas Z., Wilson J.M. (2003). Adeno-Associated Viruses Undergo Substantial Evolution in Primates during Natural Infections. Proc. Natl. Acad. Sci. USA.

[43] Smith L.J., Ul-Hasan T., Carvaines S.K., van Vliet K., Yang E., Wong K.K., Agbandje-McKenna M., Chatterjee S. (2014). Gene Transfer Properties and Structural Modeling of Human Stem Cell-Derived AAV. Mol. Ther..

[44] Sasaki N., Kok C.Y., Westhaus A., Alexander I.E., Lisowski L., Kizana E. (2023). In Search of Adeno-Associated Virus Vectors with Enhanced Cardiac Tropism for Gene Therapy. Heart Lung Circ..

[45] Asokan A., Shen S. (2023). Redirecting AAV Vectors to Extrahepatic Tissues. Mol. Ther..

[46] Zengel J., Carette J.E., Kielian M., Mettenleiter T.C., Roossinck M.J. (2020). Chapter Two-Structural and Cellular Biology of Adeno-Associated Virus Attachment and Entry. Advances in Virus Research.

[47] Wang L., Bell P., Somanathan S., Wang Q., He Z., Yu H., McMenamin D., Goode T., Calcedo R., Wilson J.M. (2015). Comparative Study of Liver Gene Transfer with AAV Vectors Based on Natural and Engineered AAV Capsids. Mol. Ther. J. Am. Soc. Gene Ther..

[48] Cabanes-Creus M., Hallwirth C.V., Westhaus A., Ng B.H., Liao S.H.Y., Zhu E., Navarro R.G., Baltazar G., Drouyer M., Scott S. (2020). Restoring the Natural Tropism of AAV2 Vectors for Human Liver. Sci. Transl. Med..

[49] Zeissler D., Busch M., Grieskamp S., Meinhardt E., Wurzer H., Kulkarni S.R., Schrattel A.K., Molter S., Kehr D., Qatato M. (2025). Novel Human Heart–Derived Natural Adeno-Associated Virus Capsid Combines Cardiospecificity with Cardiotropism In Vivo. Circulation.

[50] Summerford C., Samulski R.J. (1998). Membrane-Associated Heparan Sulfate Proteoglycan Is a Receptor for Adeno-Associated Virus Type 2 Virions. J. Virol..

[51] Opie S.R., Warrington K.H., Agbandje-McKenna M., Zolotukhin S., Muzyczka N. (2003). Identification of Amino Acid Residues in the Capsid Proteins of Adeno-Associated Virus Type 2 That Contribute to Heparan Sulfate Proteoglycan Binding. J. Virol..

[52] Qing K., Mah C., Hansen J., Zhou S., Dwarki V., Srivastava A. (1999). Human Fibroblast Growth Factor Receptor 1 Is a Co-Receptor for Infection by Adeno-Associated Virus 2. Nat. Med..

[53] Summerford C., Bartlett J.S., Samulski R.J. (1999). αVβ5 Integrin: A Co-Receptor for Adeno-Associated Virus Type 2 Infection. Nat. Med..

[54] Asokan A., Hamra J.B., Govindasamy L., Agbandje-McKenna M., Samulski R.J. (2006). Adeno-Associated Virus Type 2 Contains an Integrin α5β1 Binding Domain Essential for Viral Cell Entry. J. Virol..

[55] Pillay S., Meyer N.L., Puschnik A.S., Davulcu O., Diep J., Ishikawa Y., Jae L.T., Wosen J.E., Nagamine C.M., Chapman M.S. (2016). An Essential Receptor for Adeno-Associated Virus Infection. Nature.

[56] Uhlén M., Fagerberg L., Hallström B.M., Lindskog C., Oksvold P., Mardinoglu A., Sivertsson Å., Kampf C., Sjöstedt E., Asplund A. (2015). Proteomics. Tissue-Based Map of the Human Proteome. Science.

[57] Tabula Sapiens Consortium (2022). The Tabula Sapiens: A Multiple-Organ, Single-Cell Transcriptomic Atlas of Humans. Science.

[58] Chen C.-L., Jensen R.L., Schnepp B.C., Connell M.J., Shell R., Sferra T.J., Bartlett J.S., Clark K.R., Johnson P.R. (2005). Molecular Characterization of Adeno-Associated Viruses Infecting Children. J. Virol..

[59] Cabanes-Creus M., Westhaus A., Navarro R.G., Baltazar G., Zhu E., Amaya A.K., Liao S.H.Y., Scott S., Sallard E., Dilworth K.L. (2020). Attenuation of Heparan Sulfate Proteoglycan Binding Enhances In Vivo Transduction of Human Primary Hepatocytes with AAV2. Mol. Ther. Methods Clin. Dev..

[60] Schnepp B.C., Jensen R.L., Clark K.R., Johnson P.R. (2009). Infectious Molecular Clones of Adeno-Associated Virus Isolated Directly from Human Tissues. J. Virol..

