# Supplementary material for: Mapping of Pre-Existing Humoral Immunity to AAV and Natural AAV Capsid Presence in Human Tissues: Observations from a Polish Surgical Cohort
Source: Viruses. 2026 Oct 13;18(10):1134. doi: 10.3390/v18101134 (PMC13709496; doi:10.3390/v18101134)
Supplement: Supplementary file 1 [file viruses-18-01134-s001.zip › viruses-4380667-supplementary.pdf]

# Mapping of pre-existing humoral immunity to AAV and natural AAV capsid presence in human tissues: observations from a Polish surgical cohort.

**Aleksandra Majewska, Klara Weronika Siejda, Maciej Kowalec, Karolina Aleksandrowicz, Yuliia Panchuk, Małgorzata Marta Rogala, Karolina Pianka, Aleksandra Minich, Weronika Jarocka, Maciej Walędziak, Katarzyna Komorowska, Andrzej Kwiatkowski, Sylwia Rydzik, Tomasz Syryło, Agnieszka Bajek, Michał Wiłkojć, Leszek Gryszko, Grzegorz Suwalski, Jakub Mróz, Michał Zembala, Jacek Doniec, Klaudia Klarysa Brodaczewska and Leszek Lisowski**

## Supplementary Materials and Methods

**Table S1**. Nucleotide sequences of primers used for PCR reaction

| Primers (name_sequence) | Published |
|---|---|
| Forward | |
| AAV_1202_5'-GGTACCAAAACAAATGTTCTCGTCACG-3' | not published |
| 19S_5'-GGTAATGCCTCGGGAAATTGGCATT-3' | Gao et al., 2002 |
| | |
| Reverse | |
| AAV_1203_5'-GGAGTGG**C**CAACTCCATCACTAG-3' | not published |
| AAV_1204_5'-GGAGTGG**G**CAACTCCATCACTAG-3' | not published |
| 18aS_5'-GAATCCCCAGTTGTTGTTGATGAGTC-3' | Gao et al., 2002 |

## Supplementary Figures

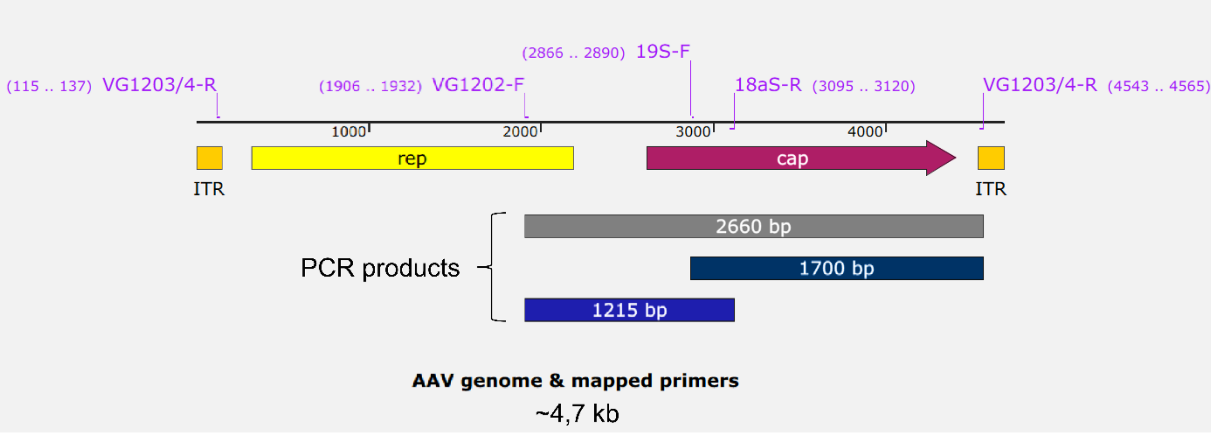


**Figure S1**. AAV genome simple graph based on AAV2 genome (NC_001401.2) with mapped ITR, rep, cap, primer targets and amplicon size. Prepared with use of SnapGene (Dotmatics) software.

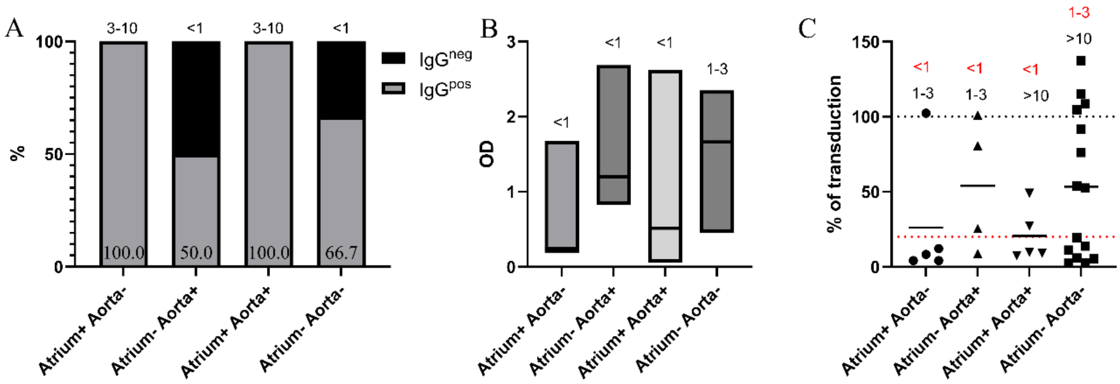


**Figure S2.** Immune responses in cardiovascular tissue patients, stratified into groups based on AAV PCR-positivity. (A) Percentage of seropositive samples in cardiovascular specimen group, divided into groups based on AAV PCR-positivity. Values above bars are $BF_{10}$ in Bayesian Binomial Test, compared to cardio group mean seropositivity (50%). (B) Level of anti-AAV2 antibodies (OD values) in cardiovascular group, divided into groups based on AAV PCR-positivity. Values above bars are $BF_{10}$ in Bayesian Wilcoxon Signed-Rank Test, compared to cardio group mean OD (1.21). (C) Percentage of AAV-mediated transduction efficiency in HEK293T cells in the presence of patient sera, reflecting neutralizing antibody activity. Values above bars are $BF_{10}$ in Bayesian Wilcoxon Signed-Rank Test, compared to: black- control (100%), red- cardio group mean % of transduction change (20%). Due to small group sizes, statistical analysis was performed using Bayesian models in JASP software (ASP Team (2026). JASP (Version 0.98.0). Atrium+ Aorta- n=5, Atrium- Aorta+ n=4, Atrium+ Aorta+ n=5, Atrium- Aorta- n=15.

$BF_{10}$<1 evidence for no effect, $BF_{10}$ 1-3 anecdotal evidence for an effect, $BF_{10}$ 3-10 moderate evidence for an effect, $BF_{10}$>10 strong evidence for an effect
